# Development, validation, and visualization of a web-based nomogram to predict 5-year mortality risk in older adults with hypertension

**DOI:** 10.1186/s12877-022-03087-3

**Published:** 2022-05-04

**Authors:** Huanrui Zhang, Wen Tian, Yujiao Sun

**Affiliations:** Department of Geriatrics, The First Affiliated Hospital of China Medical University, No.155 Nanjing North Street, Shenyang, 110001 China

**Keywords:** Hypertension, Mortality, Older, Nomogram, Prediction model

## Abstract

**Background:**

Hypertension-related mortality has been increasing in older adults, resulting in serious burden to society and individual. However, how to identify older adults with hypertension at high-risk mortality remains a great challenge. The purpose of this study is to develop and validate the prediction nomogram for 5-year all-cause mortality in older adults with hypertension.

**Methods:**

Data were extracted from National Health and Nutrition Examination Survey (NHANES). We recruited 2691 participants aged 65 years and over with hypertension in the NHANES 1999-2006 cycles (training cohort) and 1737 participants in the NHANES 2007-2010 cycles (validation cohort). The cohorts were selected to provide at least 5 years follow-up for evaluating all-cause mortality by linking National Death Index through December 31, 2015. We developed a web-based dynamic nomogram for predicting 5-year risk of all-cause mortality based on a logistic regression model in training cohort. We conducted internal validation by 1000 bootstrapping resamples and external validation in validation cohort. The discrimination and calibration of nomogram were evaluated using concordance index (C-index) and calibration curves.

**Results:**

The final model included eleven independent predictors: age, sex, diabetes, cardiovascular disease, body mass index, smoking, lipid-lowering drugs, systolic blood pressure, hemoglobin, albumin, and blood urea nitrogen. The C-index of model in training and validation cohort were 0.759 (bootstrap-corrected C-index 0.750) and 0.740, respectively. The calibration curves also indicated that the model had satisfactory consistence in two cohorts. A web-based nomogram was established (https://hrzhang1993.shinyapps.io/dynnomapp).

**Conclusions:**

The novel developed nomogram is a useful tool to accurately predict 5-year all-cause mortality in older adults with hypertension, and can provide valuable information to make individualized intervention.

**Supplementary Information:**

The online version contains supplementary material available at 10.1186/s12877-022-03087-3.

## Background

Currently, the global aging of population has become serious, it is predicted that the global population of older persons is supposed to reach more than two billion in 2050 [1]. The prevalence of hypertension increased with age, the prevalence of hypertension was found over 60% for those aged ≥60 years and 74% for those aged ≥80 years [2, 3]. Hypertension and its associated diseases, including cardiovascular disease [4], kidney disease [5], pulmonary disease [6], neurological disease [7], infectious disease [8], and cancer [9], seriously affect the health and living quality of human being, thus induce higher mortality. Epidemiological studies have demonstrated that hypertension-related mortality has been continuously rising, particularly among older adults [10, 11]. The profound impact of hypertension on mortality brings a heavy burden to families and society [12].

Early identifying those at high-risk mortality, to take timely interventions, would help to reduce premature mortality risk in older adults with hypertension. Therefore, it is highly necessary to work out a mortality prediction model for older adults with hypertension, only a few studies developed a prediction model of mortality for the hypertension population [13–15]. However, those studies were limited by their study population, follow-up time and risk calculation model of mortality, failed to extend those models to general older adults with hypertension. To date, there is no population-based study to construct a risk prediction model of mortality in older adults with hypertension. Nomogram is a visual statistical prognostic tool, which is widely applied in prognostic evaluation of clinical outcomes by calculating a score to potential predictors [16]. Nomogram is a simple, effective and reliable prediction model to quickly provide clinical risk stratification and prognosis decision. The present study is to develop and validate the prediction nomogram for 5-year all-cause mortality in older adults with hypertension based on a nationally representative population in US. 

## Methods

### Study design and participants

**Fig. 1:**
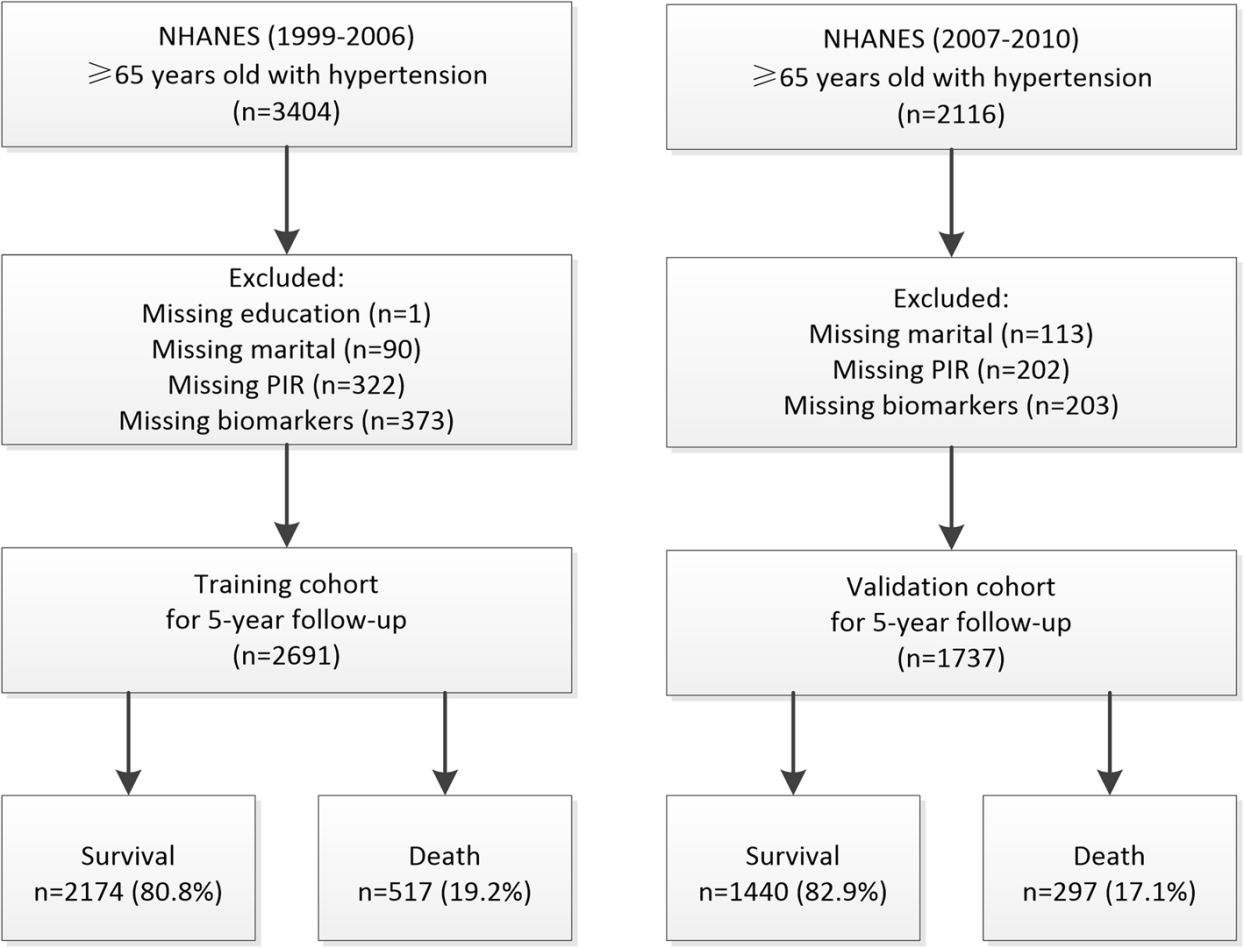
Flow chart of the training and validation cohorts

Data were extracted from the National Health and Nutrition Examination Survey (NHANES), a series of complex, stratified, multistage sampling design aimed to assess the health status of citizens in the US. All the participants signed informed consent and the survey protocols were ratified by the Research Ethics Review Board of the National Center for Health Statistics. And all procedures were performed in accordance with relevant guidelines and regulations. For this study, we selected the participants in the NHANES 1999-2006 cycles as training cohort, while the validation cohort was comprised of participants in the NHANES 2007-2010 cycles. We included participants aged 65 years or older with hypertension in baseline. The definition of hypertension was self-reported hypertension, SBP ≥140 mmHg, or DBP ≥90 mmHg or reported use of antihypertensive [17]. Participants without information on follow-up outcome and key candidate variables were excluded. The detailed selection process was shown in Fig. 1. The follow-up all-cause mortality was determined by the linked National Death Index through December 31, 2015. The training and validation cohort were selected to provide at least 5-year follow-up for evaluating all-cause mortality.

### Potential predictors

We included various predictors that have been linked to death in patients with hypertension or older adults in former studies through face-to-face interviews, physical examination and laboratory tests. These predictors included demographics (age, sex, ethnicity, education, marital status, and the income to poverty ratios), smoking, comorbidities (cardiovascular disease and diabetes), prescriptions (antihypertensive, hypoglycemic, lipid-lowering, and antiplatelet drugs) and biomarkers such as Body mass index (BMI), systolic (SBP) and diastolic (DBP) blood pressure, total cholesterol/high-density lipoprotein cholesterol ratio (TC/HDLC), hemoglobin A1C (HbA1C), white blood cells, hemoglobin, platelet, albumin, blood urea nitrogen (BUN), estimated glomerular filtration rate (eGFR). HDL-C was obtained by direct immunoassay or a heparin-manganese precipitation method, while TC was measured by an enzymatic assay method. HbA1C measurements were performed on the Glycohemoglobin Analyzer. Hemoglobin, white blood cells, and platelet counts were evaluated using automated hematology analyzing devices. Albumin concentration was measured by a bichromatic digital endpoint method using the DxC800 modular chemistry. The concentration of Bun was determined by means of the enzymatic conductivity rate method using The DxC800 modular chemistry (BUNm). Serum creatinine was measured by the Jaffe rate method (kinetic alkaline picrate) using the DxC800 modular chemistry side. We categorized ethnicity into four groups (Non-Hispanic White, Non-Hispanic Black, Other Hispanic, and other race), education in two levels (less than high school and high school or above), marital status in three groups (married, widowed or divorced, and single), the income to poverty ratios (IPR) in two levels (IPR ≤ 1.3 and IPR > 1.3) and smokers in three groups (never, former and current) [18]. Cardiovascular disease (CVD) included congestive heart failure, coronary heart disease, angina pectoris, heart attack or stroke. The definition of diabetes was self-reported diabetes, HbA1C ≥ 6.5%, fasting plasma glucose level ≥ 126 mg/dL, or the use of hypoglycemic [19]. BMI was computed as weight in kilograms divided by the square of height in meters. The eGFR was derived from the Chronic Kidney Disease Epidemiology equation [20].

### Statistical analyses

All statistical analyses were conducted using R version 4.0.3 statistical software and significance was set a priori at *p* < 0.05 with two-sided. Continuous variables were described as mean and standard deviation (SD), and categorical variables were expressed as count and percentage. The odds ratio (OR) and 95% confidential interval (CI) were estimated for every variable using the logistic regression model. To identify the final prediction model, a backward stepwise selection method with the Akaike information criterion (AIC) was performed to select the best potential predictive variables in multivariable logistic regression. The nomogram was depicted based on the final prediction model using the “rms” package. To test the model’s performance, internal validation by bootstrapping with 1000 resamples and external validation were performed in the training and validation cohort, respectively. We estimated the model’s performance by measuring the discrimination and calibration. Discrimination efficiency refers to the capacity of a prediction model distinguishes between patients with and without the outcome. The concordance index (C-index) was applied to evaluate the discrimination and C-index ≥0.7 was defined as having good discrimination [21]. The calibration refers to the agreement between the predicted outcomes and the actual outcomes. The calibration curve was depicted to evaluate the calibration and the curve closed to the diagonal line was seen as having perfect calibration. Meanwhile, a dynamic nomogram was constructed for conveniently predicting the 5-year risk of all-cause mortality among older adults with hypertension on the website (https://hrzhang1993.shinyapps.io/dynnomapp).

## Results

### Baseline characteristics

The final study included 2691 participants in training cohort and 1737 participants in validation cohort. There were 517 (19%) and 297 (17%) deaths during the 5-year follow-up period in training and validation cohort, respectively. Descriptive statistics of two cohorts are shown in Table 1. The all-cause mortality of the two cohorts by sex and age are presented in Table 2.

**Table 1:** Baseline characteristics in training and validation cohorts

Variables	training cohort	validation cohort
	(*n* = 2691)	(*n* = 1737)
Demographic		
Age, years	74.70 ± 6.41	73.74 ± 5.17
Sex, n (%)		
Male	1311 (48.7)	848 (48.8)
Female	1380 (51.3)	889 (51.2)
Ethnicity, n (%)		
Non-Hispanic White	1675 (62.2)	1090 (62.8)
Non-Hispanic Black	453 (16.8)	286 (16.5)
Other Hispanic	75 (2.8)	119 (6.9)
Other races	488 (18.1)	242 (13.9)
Education, n (%)		
less than high-school	1111 (41.3)	631 (36.3)
high school or above	1580 (58.7)	1106 (63.7)
Marital status, n (%)		
Married	1477 (54.9)	958 (55.2)
Widowed or divorced	1099 (40.8)	694 (40.0)
Single	115 (4.3)	85 (4.9)
The income to poverty ratios, n (%)		
≤1.3	1885 (70.0)	1237 (71.2)
> 1.3	806 (30.0)	500 (28.8)
Smoking, n (%)		
Never	1312 (48.8)	817 (47.0)
Former	1152 (42.8)	759 (43.7)
Current	227 (8.4)	161 (9.3)
Comorbidities		
Cardiovascular disease, n (%)		
Absence	1890 (70.2)	1216 (70.0)
Presence	801 (29.8)	521 (30.0)
Diabetes, n (%)		
Absence	1979 (73.5)	1175 (67.6)
Presence	712 (26.5)	562 (32.4)
Biomeasures		
Body mass index, kg/m2, n (%)		
< 25	735 (27.3)	435 (25.0)
25-30	1084 (40.3)	652 (37.5)
≥30	872 (32.4)	650 (37.4)
Systolic blood pressure, mmHg	148.97 ± 22.69	142.07 ± 21.56
Diastolic blood pressure, mmHg	69.90 ± 13.73	67.07 ± 13.34
Total cholesterol /High density lipoprotein cholesterol	4.07 ± 1.32	3.85 ± 1.32
White blood cells, 1000 cells/uL	7.14 ± 2.88	7.07 ± 2.21
Hemoglobin, g/dL	14.06 ± 1.45	13.81 ± 1.53
Platelet, 1000 cells/uL	251.01 ± 70.90	237.57 ± 69.72
Albumin, g/dL	41.91 ± 3.03	41.55 ± 3.02
Blood urea nitrogen, mg/dL	6.28 ± 2.84	6.33 ± 2.90
eGFR, mg/min/1.73 m2	69.94 ± 20.92	68.53 ± 19.52
HbA1C, %	5.92 ± 1.02	6.06 ± 0.93
Drugs		
Antihypertensive drugs, n (%)		
Absence	963 (35.8)	421 (24.2)
Presence	1728 (64.2)	1316 (75.8)
Hypoglycemic agents, n (%)		
Absence	2288 (85.0)	1483 (85.4)
Presence	403 (15.0)	254 (14.6)
Lipid-lowering drugs, n (%)		
Absence	1898 (70.5)	1016 (58.5)
Presence	793 (29.5)	721 (41.5)
Antiplatelet drugs, n (%)		
Absence	2550 (94.8)	1515 (87.2)
Presence	141 (5.2)	222 (12.8)
5-year mortality, n (%)		
Survival	2174 (80.8)	1440 (82.9)
Death	517 (19.2)	297 (17.1)

*HbA1C* hemoglobin, *eGFR* estimated glomerular filtration rate

**Table 2:** Mortality in the training and validation cohorts

	Training cohort		Validation cohort	
Variables	Suvival	Death	Suvival	Death
	(*n* = 2174)	(*n* = 517)	(*n* = 1440)	(*n* = 297)
Sex, n (%)				
Male	1007 (46.3)	304 (58.8)	682 (47.4)	166 (55.9)
Female	1167 (53.7)	213 (41.2)	758 (52.6)	131 (44.1)
Age, n (%)				
65~	650 (29.9)	63 (12.2)	415 (28.8)	41 (13.8)
70~	598 (27.5)	98 (19.0)	426 (29.6)	52 (17.5)
75~	394 (18.1)	103 (19.9)	293 (20.3)	57 (19.2)
80~	532 (24.5)	253 (48.9)	306 (21.2)	147 (49.5)

### Predictors of mortality

We found that most potential predictors were associated with mortality except education, IPR, antihypertensive drugs, TC/HDLC, HbA1C, and platelet in univariable logistic regression model (Supplementary Table 1). The full model constructed via all candidate predictors was presented in Table 3. Meanwhile, the final model constructed by a backward stepwise selection method with the Akaike information criterion (AIC) was also shown in Table 3. Hosmer-Lemeshow test (*P* > 0.05) and C-index indicated both the full model and the final model have good model performance. Therefore, the simpler final model was applied for further nomogram plotting. The final model included eleven independent predictors: age, sex, diabetes, cardiovascular disease, BMI, smoking, lipid-lowering drugs, SBP, hemoglobin, albumin, and BUN.

**Table 3:** Multivariable logistic regression analysis in the training cohorts

Variables	Full Model^a^		Final Model^b^	
	OR (95% *CI*)	*P* value	OR (95% *CI*)	*P* value
Factors Selected				
Age, years	1.101 [1.079, 1.124]	< 0.001	1.105 [1.085, 1.126]	< 0.001
Sex				
Male	Ref.		Ref.	
Female	0.452 [0.346, 0.587]	< 0.001	0.504 [0.397, 0.639]	< 0.001
Diabetes				
Absence	Ref.		Ref.	
Presence	1.409 [0.982, 2.007]	0.060	1.415 [1.112, 1.798]	0.005
Cardiovascular disease				
Absence	Ref.		Ref.	
Presence	1.821 [1.443, 2.296]	< 0.001	1.814 [1.449, 2.268]	< 0.001
Body mass index, kg/m2				
< 25	Ref.		Ref.	
25-30	0.728 [0.562, 0.942]	0.016	0.714 [0.554, 0.919]	0.009
≥30	0.737 [0.549, 0.990]	0.043	0.733 [0.550, 0.976]	0.034
Smoking				
Never	Ref.		Ref.	
Former	1.315 [1.043, 1.659]	0.021	1.313 [1.043, 1.653]	0.021
Current	2.459 [1.653, 3.635]	< 0.001	2.687 [1.830, 3.921]	< 0.001
Lipid-lowering drugs				
Absence	Ref.		Ref.	
Presence	0.676 [0.520, 0.873]	0.003	0.672 [0.522, 0.861]	0.002
Systolic blood pressure, mmHg	1.005 [1.000, 1.010]	0.061	1.005 [1.000, 1.010]	0.030
Hemoglobin, g/dL	0.898 [0.823, 0.981]	0.017	0.904 [0.833, 0.981]	0.016
Albumin, g/dL	0.926 [0.892, 0.962]	< 0.001	0.924 [0.890, 0.958]	< 0.001
Blood urea nitrogen, mg/dL	1.083 [1.037, 1.131]	< 0.001	1.087 [1.050, 1.125]	< 0.001
Factors Not Selected				
Ethnicity				
Non-Hispanic White	Ref.		Ref.	
Non-Hispanic Black	0.875 [0.628, 1.213]	0.427	NA	NA
Other Hispanic	0.556 [0.240, 1.157]	0.139	NA	NA
Other races	0.878 [0.627, 1.220]	0.444	NA	NA
Education				
less than high school	Ref.		Ref.	
high school or above	0.942 [0.743, 1.196]	0.624	NA	NA
The income to poverty ratios				
≤1.3	Ref.		Ref.	
> 1.3	1.156 [0.903, 1.477]	0.248	NA	NA
Diastolic blood pressure, mmHg	1.003 [0.994, 1.011]	0.528	NA	NA
Antihypertensive drugs				
Absence	Ref.		Ref.	
Presence	0.970 [0.765, 1.230]	0.798	NA	NA
Hypoglycemic agents				
Absence	Ref.		Ref.	
Presence	1.141 [0.763, 1.714]	0.522	NA	NA
Antiplatelet drugs				
Absence	Ref.		Ref.	
Presence	0.841 [0.537, 1.294]	0.440	NA	NA
Marital status				
Married	Ref.		Ref.	
Widowed or divorced	1.230 [0.963, 1.572]	0.097	NA	NA
Single	1.041 [0.586, 1.776]	0.886	NA	NA
Total cholesterol /High density lipoprotein cholesterol	1.005 [0.925, 1.091]	0.901	NA	NA
White blood cells, 1000 cells/uL	1.020 [0.984, 1.054]	0.213	NA	NA
HbA1C, %	0.952 [0.826, 1.091]	0.490	NA	NA
Platelet, 1000 cells/uL	1.001 [0.999, 1.002]	0.460	NA	NA
eGFR, mg/min/1.73 m2	0.999 [0.992, 1.006]	0.788	NA	NA
Model performance	Full Model		Final Model	
AIC	2305.8		2286.0	
C-index (95%CI)	0.762(0.739-0.784)		0.759(0.736-0.782)	
Hosmer-Lemeshow test^c^	χ2 = 6.065 (P value =0.640)		χ2 = 10.976 (P value =0.203)	

^a^The full model included 24 predictors: age, sex, ethnicity, education, marital status, the income to poverty ratios, smoking, cardiovascular disease, diabetes, antihypertensive, hypoglycemic, lipid-lowering, and antiplatelet drugs, BMI, SBP, DBP, TC/HDLC, HbA1C, white blood cells, hemoglobin, platelet, albumin, BUN, and eGFR

^b^The final model included 11 predictors: age, sex, cardiovascular disease, diabetes, BMI, smoking, lipid-lowering drugs, SBP, hemoglobin, albumin, and BUN

^c^The Hosmer-Lemeshow test was applied to test the goodness of fit and a *P* value > 0.05 was considered good fit *HbA1C* hemoglobin*, eGFR* estimated glomerular filtration rate, *CI* confidence interval, AIC Akaike information criterion

### Development of nomogram

**Fig. 2:**
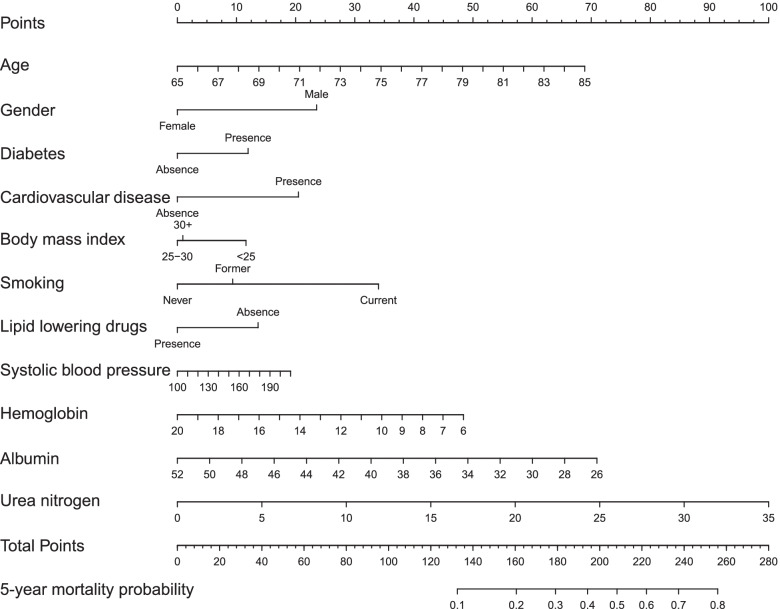
Nomogram for estimating the 5-year all-cause mortality probability. The mortality risk nomogram was constructed using the predictors, including age, sex, cardiovascular disease, diabetes, body mass index, smoking, lipid-lowering drugs, systolic blood pressure, hemoglobin, albumin, and blood urea nitrogen

Based on the results from the final model, a nomogram was constructed for forecasting the 5-year all-cause mortality probability of older adults with hypertension (Fig. 2). The nomogram contained 14 axes and axis 2-12 represented each prognostic factor of the final model. Each predictor was assigned a different weighted score in the nomogram. And, axis 13-14 implied that the higher total points were related to higher mortality risk.

### Internal and external validation

**Fig. 3:**
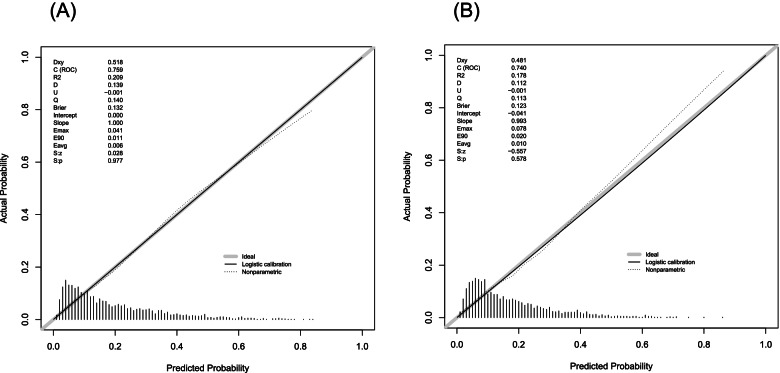
Calibration curves for the monogram in cohorts. **A** Training cohort. **B** validation cohort. The x- and y- axis represents the predicted probability and the actual observed probability of 5-year all-cause mortality, respectively

In the training cohort, the unadjusted C-index (0.759) and bootstrap-corrected C-index (0.750) indicated that the model had good discrimination. The calibration curve closed to the diagonal line indicated the model had satisfactory consistency between the predicted outcomes and the actual outcomes (Fig. 3a). In the validation cohort, there was also good discrimination for the model with the C-index of 0.740. Moreover, the calibration plot also implied that the model had an adequate fit for 5-year all-cause mortality (Fig. 3b).

### Online dynamic Nomogram

**Fig. 4:**
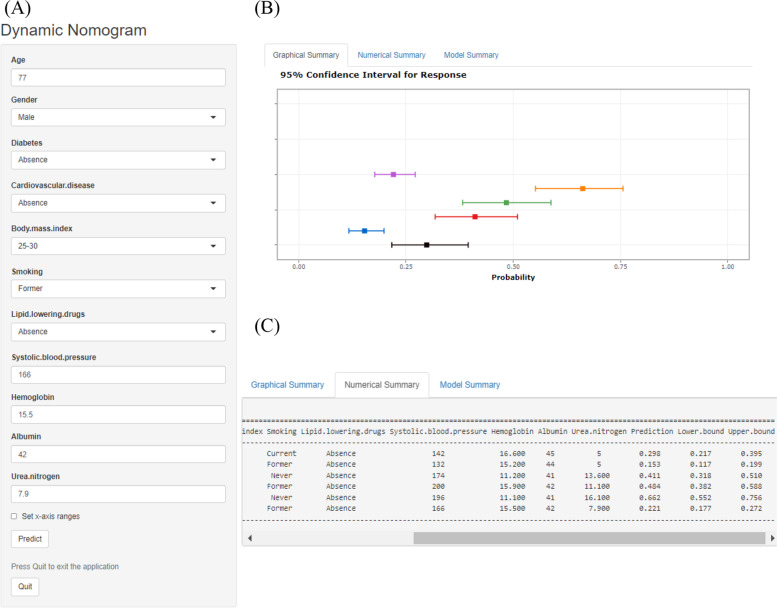
Web-based dynamic nomogram for prediction 5-year all-cause mortality probability. By entering the specific information of the older adult with hypertension in the web-online tool, we could obtain the participant’s corresponding 5-year all-cause mortality probability. **A** Entering Interface: You can enter the specific information of the participant in this interface. **B** Graphical Summary: The 5-year all-cause mortality probability and 95% confidence interval of participants are depicted in this interface. **C** Numerical Summary: The actual values of 5-year all-cause mortality probability and 95% confidence interval are shown in this interface

To conveniently predict the 5-year risk of all-cause mortality in older adults with hypertension in daily use, we developed a dynamic nomogram on the website (https://hrzhang1993.shinyapps.io/dynnomapp). By entering the specific information of the older adult in the web-online tool, we could obtain the 5-year all-cause mortality probability for the older adult (Fig. 4).

## Discussion

In the NHANES follow-up cohort, our study developed and validated a novel and practical nomogram model to predict 5-year all-cause mortality risk of older adults with hypertension. Eleven available predictors for 5-year mortality nomogram, including age, sex, diabetes, CVD, BMI, smoking, lipid-lowering drugs, SBP, hemoglobin, albumin and BUN, were identified by logistic regression model. By analyzing C-index and calibration curve in the internal and external validation cohorts, the novel nomogram exhibited favorable predictive performance and stability. For convenience, we constructed the personalized and user-friendly web-based nomogram, freely available online (http://hrzhang1993.shinyapps.io/dynnomapp), to provide an individualized 5-year all-cause mortality probability and help physicians to make earlier individualized intervention in older adults with hypertension.

The prognosis of various diseases has been increasingly assessed with nomogram in recent years, including cancer [22], renal failure [23], myocardial infarction [24], and pancreatitis [25]. As the prolonged of life expectancy, the proportion of older adults with hypertension is increasing. A universal risk assessment tool of hypertension-related all-cause mortality in older adults is essential. However, no previous studies have developed the nomogram for predicting 5-year all-cause mortality in older adults with hypertension. The prognostic nomogram, involving demographic characteristics and clinical routine laboratory parameters, was constructed in the study, which could provide important prognostic information for making rational individual intervention strategies to decline the risk of premature mortality in older adults with hypertension.

Several kinds of the mortality prediction models for people with hypertension have been constructed in previous studies. A prediction model (points system and mobile application) was developed to identify the risk of one-year mortality in hypertensive patients admitted through the emergency department in Spain [15]. Its model variables included the charlson comorbidity index, self-care and usual activities derived from the EuroQol five dimensions questionnaire, which were difficult to achieve in the general population. The one-year mortality prediction model of hypertensive inpatients with strict limitations was quite different from our study. Subjects in the other two studies were from clinical trials [13, 14]. Clinical trials have strict inclusion and exclusion criteria, which lead to selection bias, so their model does not apply to the general population [26]. Pocock et al. established a 5-year cardiovascular mortality prediction model, which must be calculated using a mathematical formula [13]. The 10-year death probability was not achieved in their prediction model by huynt et al. [14]. Our study population came from a representative of the general US population, so our model was characterized with a wider range of its availability. As a widely used prediction tool, nomogram is a graphic predictive model that provides real-time and accurate personalized risk stratification. So, we created a 5-year all-cause mortality risk prediction nomogram for risk stratification of older adults with hypertension.

Of all 24 potential variables, 11 valuable predictive factors were involved in our nomogram by multivariable logistic regression to predict 5-year mortality in older adults with hypertension. Aging is an inevitable process of functional impairment and increases the susceptibility to a variety of age-dependent diseases, ultimately leading to death [27]. Age is a known and important risk factor for mortality. It is widely known that mortality increases exponentially with age, which usually happens among people age 35 or older [28, 29]. In general, the life expectancy of male is shorter than that of female. The life expectancy of male was 76.2 years, and female was 81.2 years in 2018 for the US population [30]. A higher mortality has been reported in male than female [31]. Male has always been a risk factor for cardiovascular diseases [32]. Besides sex-specific cancer, the prevalence in male and female is > 1 for all cancers, and the cancer-related burden is higher in male than female [33]. Heart disease, stroke and diabetes were the first, fifth and seventh leading causes of death in the US, respectively [30]. Age, sex, CVD and diabetes can be used as non-modifiable risk factors for predicting the risk of 5-year mortality in older adults with hypertension, consistent with previous studies.

In our prediction nomogram, the other seven predictors could be modified. The effective management and intervention of these modifiable prediction factors will be of significance in reducing the risk of mortality among older adults with hypertension. A meta-analysis focused on ≥65 years older adults, found a U-shaped association between BMI and mortality and the lowest risk of mortality in those with BMI from 24 to 30.9 [34]. Older adults with underweight had a higher risk of mortality [35, 36], which might be related to malnutrition and chronic diseases in those people [37]. A similar trend was observable in the study, older adults with BMI < 25 kg/m^2^ had the highest risk score of mortality. Smoking increased significantly risks of most diseases, such as various cancers, respiratory diseases, and CVD, eventually causing premature death [38]. Smoking is one of the main causes of preventable death in the US [39]. Smoking cessation reduces mortality risk and extends life expectancy [40]. In the nomogram, the risk score of mortality decreased noticeably after quitting smoking, the positive and effective response measures should be taken to stop smoking.

Statins are the most commonly used lipid-lowering drug worldwide to manage dyslipidemia and CVD [41]. Statins have long been the cornerstone of CVD therapy to reduce CVD death [42]. A recent meta-analysis found statins significantly reduced the risk of all-cause mortality in older adults [43, 44], consisted with our results in terms of the benefit of lipid-lowering therapy. A U-shaped or J-shaped association between SBP and mortality has been found [45]. In the older population, a U-shaped relationship between SBP and mortality was found [46, 47]. CLHLS study showed the lowest risk of mortality was found in older adults with a range of SBP 107-154 mmHg, lower and higher SBP increased the risk of mortality [46]. However, our study population was older adults with hypertension, only a very small percentage of older adults had lower SBP, so the lowest SBP was 90 mmHg in the prediction model and the risk score of mortality increased with SBP. Hemoglobin decreased and anemia increased with age [48]. There existed a dose-dependent relationship between the severity of anemia and all-cause mortality in older adults [49, 50]. Albumin is viewed as a nutrition indicator to assess the nutrition status in older adults, and also a marker of inflammation [51]. The albumin level reduced 0.08-0.17 g/l per year with age [52], and predicted the risk of mortality [53]. Studies indicated hypoalbuminemia usually occurred concomitantly with anemia in older adults [51, 54]. Anemia and hypoalbuminemia were both associated with malnutrition and frailty, and considered to be independent risk factors for mortality in older adults [54]. Our nomogram showed that the levels of hemoglobin and albumin had negative association with the risk score of mortality. BUN is a sensitive index for reflecting renal function, many studies suggested BUN was associated with increased all-cause mortality in various populations [55, 56]. BUN could reflect the whole health status and functional level in the general older population [55]. Our study showed BUN was an independent risk factor for 5-year all-cause mortality in older adults with hypertension. In our nomogram, the risk of 5-year mortality was identified by analyzing the potential predictor in older adults with hypertension, it is of great significance to aggressive control of these modifiable risk factors for reducing the risk of mortality.

A few limitations existed in the study. First, the study population was from the general US population, the mortality prognostic nomogram might not be generalized to non-US population and critically ill patients with hypertension. Second, the prediction model was designed for older adults with hypertension, our results could not be applied to individuals under 65 years old. Finally, some other unknown confounding factors related to mortality might be excluded from our model. Despite its limitations, the predictive nomogram was constructed by rigorous methods, including selection of predictors, internal and external validation, could provide a reliable and accurate risk stratification tool of mortality in older adults with hypertension.

## Conclusions

In conclusion, the study developed, validated, and visualized a novel web-based nomogram to predict 5-year all-cause mortality in older adults with hypertension, consisting of 11 common clinical characteristics. The nomogram provided a satisfactory predictive performance to identify the high-risk group of 5-year all-cause mortality, which would help to determine aggressive individualized treatment for reducing the risk of premature mortality among older adults with hypertension. Future prospective and interventional studies will be needed to confirm our nomogram.

## Supplementary Information

**Additional file 1.**

## Data Availability

All data were included in NHANES database (https://www.cdc.gov/nchs/nhanes/ index.htm).
