# Supplementary material for: Development, validation, and visualization of a web-based nomogram to predict 5-year mortality risk in older adults with hypertension
Source: BMC Geriatr. 2022 May 4;22:392. doi: 10.1186/s12877-022-03087-3 (PMC9069777; doi:10.1186/s12877-022-03087-3)
Supplement: Supplementary file 1 — Additional file 1. [file 12877_2022_3087_MOESM1_ESM.docx]

| Supplementary table 1 Univariable logistic regression analysis in the Training Cohorts | | |
| --- | --- | --- |
| Variables | OR (95% CI) | P value |
| Age, years | 1.108 (1.090~1.126) | <0.001 |
| Gender |  |  |
| Male | Ref. |  |
| Female | 0.605 (0.497~0.734) | <0.001 |
| Ethnicity |  |  |
| Non-Hispanic White | Ref. |  |
| Non-Hispanic Black | 0.906 (0.694~1.173) | 0.462 |
| Other Hispanic | 0.520 (0.239~1.000) | 0.070 |
| Other races | 0.671 (0.506~0.879) | 0.004 |
| Marital status |  |  |
| Married | Ref. |  |
| Widowed or divorced | 1.409 (1.157~1.714) | 0.001 |
| Single | 1.028 (0.607~1.662) | 0.913 |
| Education |  |  |
| less than high school | Ref. |  |
| high school or above | 0.842 (0.694~1.022) | 0.081 |
| The income to poverty ratios |  |  |
| ≤1.3 | Ref. |  |
| >1.3 | 1.185 (0.963~1.453) | 0.106 |
| Smoking |  |  |
| Never | Ref. |  |
| Former | 1.394 (1.137~1.709) | 0.001 |
| Current | 1.812 (1.294~2.511) | <0.001 |
| Body mass index, kg/m2 |  |  |
| <25 | Ref. |  |
| 25-30 | 0.626 (0.499~0.786) | <0.001 |
| ≥30 | 0.542 (0.423~0.693) | <0.001 |
| Systolic blood pressure, mmHg | 1.005 (1.001~1.010) | 0.012 |
| Diastolic blood pressure, mmHg | 0.991 (0.984~0.998) | 0.014 |
| Diabetes |  |  |
| Absence | Ref. |  |
| Presence | 1.332 (1.078~1.641) | 0.007 |
| Cardiovascular disease |  |  |
| Absence | Ref. |  |
| Presence | 2.160 (1.771~2.632) | <0.001 |
| Antihypertensive drugs |  |  |
| Absence | Ref. |  |
| Presence | 0.950 (0.779~1.160) | 0.611 |
| Hypoglycemic agents |  |  |
| Absence | Ref. |  |
| Presence | 1.364 (1.055~1.751) | 0.016 |
| Lipid-lowering drugs |  |  |
| Absence | Ref. |  |
| Presence | 0.719 (0.575~0.894) | 0.003 |
| Antiplatelet drugs |  |  |
| Absence | Ref. |  |
| Presence | 1.534 (1.029~2.241) | 0.031 |
| Total cholesterol /High density lipoprotein cholesterol | 0.983 (0.913~1.056) | 0.640 |
| White blood cells, 1000 cells/uL | 1.032 (1.002~1.069) | 0.050 |
| HbA1C, % | 1.023 (0.930~1.120) | 0.633 |
| Hemoglobin, g/dL | 0.841 (0.787~0.898) | <0.001 |
| Platelet, 1000 cells/uL | 1.000 (0.998~1.001) | 0.482 |
| Albumin, g/dL | 0.910 (0.882~0.939) | <0.001 |
| Blood urea nitrogen, mg/dL | 1.156 (1.120~1.194) | <0.001 |
| eGFR, mg/min/1.73 m2 | 0.978 (0.973~0.982) | <0.001 |
| CI confidence interval | | |
